# In-hospital cardiopulmonary resuscitation of patients with cirrhosis: A population-based analysis

**DOI:** 10.1371/journal.pone.0222873

**Published:** 2019-09-30

**Authors:** Lavi Oud

**Affiliations:** Division of Pulmonary and Critical Care Medicine, Department of Internal Medicine, Texas Tech University Health Sciences Center at the Permian Basin, Odessa, Texas, United States of America; Azienda Ospedaliero Universitaria Careggi, ITALY

## Abstract

**Objective:**

To examine the epidemiology and outcomes of in-hospital cardiopulmonary resuscitation (CPR) among patients with cirrhosis.

**Methods:**

We used the Texas Inpatient Public Use Data File to identify hospitalizations aged ≥ 18 years with and without cirrhosis during 2009–2014 and those in each group who have undergone in-hospital CPR. Short-term survival (defined as absence of hospital mortality or discharge to hospice) following in-hospital CPR was examined. Multivariate logistic regression modeling was used to assess the prognostic impact of cirrhosis following in-hospital CPR and predictors of short-term survival among cirrhosis hospitalizations.

**Results:**

In-hospital CPR was reported in 2,511 and 51,969 hospitalizations with and without cirrhosis, respectively. The rate of in-hospital CPR (per 1,000 hospitalizations) was 7.6 and 4.0 among hospitalizations with and without cirrhosis, respectively. The corresponding rate of in-hospital CPR among decedents was 10.7% and 13.4%, respectively. Short-term survival following in-hospital CPR among hospitalizations with and without cirrhosis was 14.9% and 27.3%, respectively, and remained unchanged over time on adjusted analyses among the former (p = 0.1753), while increasing among the latter (p = 0.0404). Cirrhosis was associated with lower odds of short-term survival following in-hospital CPR (adjusted odds ratio [aOR] 0.55 [95% CI: 0.49–0.62]). Lack of health insurance (vs. Medicare) (aOR] 0.47 [95% CI: 0.34–0.67]) and sepsis ([aOR] 0.67 [95% CI: 0.53–85]) were associated with lower odds of short-term survival following in-hospital CPR among cirrhosis hospitalizations.

**Conclusions:**

The rate of in-hospital CPR was nearly 2-fold higher among hospitalizations with cirrhosis than among those without it, though it was used more selectively among the former. Short-term survival following in-hospital CPR remained markedly lower among cirrhosis hospitalizations, while progressively improving among those without cirrhosis. Strategies to increase access to health insurance and improve early identification and control of infection should be explored in future preventive and interventional efforts.

## Introduction

Cirrhosis is increasingly prevalent in the United States (US) [1] and affected patients commonly experience decompensation events, with estimated 500,000 annual hospitalizations [2]. The annual death toll of cirrhosis in the US either as a direct of an associated cause has been estimated to exceed 70,000 patients [3].

Despite the abovementioned burdens, hospital survival of patients with cirrhosis has been improving [4], including among those with critical illness [5]. However, the latter increasingly favorable short-term outcomes were not matched by previous reports of poor hospital survival among patients with cirrhosis undergoing in-hospital cardiopulmonary resuscitation (CPR) [6–8], contrasting the progressive improvement of hospital survival in the general population [9–11].

Data are scarce on the contemporary epidemiology and outcomes of in-hospital CPR among patients with cirrhosis, with reported studies focusing on select older age groups [6], describing outcomes of care provided several decades ago [6, 12], published in a preliminary form [7], or based on small, single-center cohorts [8, 13]. It is thus unclear whether the aforementioned gains in short-term outcomes among hospitalized cirrhotic patients extend to those undergoing in-hospital CPR. We asked: 1) how often is in-hospital CPR conducted among hospitalized patients with cirrhosis? 2) what are the characteristics of patients with cirrhosis who undergo in-hospital CPR? and 3) what are the short-term outcomes following in-hospital CPR among patients with cirrhosis and what factors are associated with these outcomes?

Contemporary data addressing these latter questions can inform future preventive and interventional efforts by clinicians and healthcare systems and may provide benchmark data for performance improvement initiatives.

We conducted a population-level study of hospitalized patients with and without cirrhosis to: a) examine the comparative patterns of utilization of in-hospital CPR and its associated outcomes, overall and over time b) characterize the patients undergoing in-hospital CPR and c) determine the prognostic impact of cirrhosis following in-hospital CPR and the factors associated with short-term survival among those with cirrhosis.

## Materials and methods

This was a retrospective, population-based cohort study. Because we used a publicly available, de-identified data set, the study was determined to be exempt from formal review by the Texas Tech Health Sciences Center’s Institutional Review Board. A detailed description of the study methodology is included in the Supplementary Materials (Appendix A in S1 File).

### Data sources and study population

We used the Texas Inpatient Public Use Data File (TIPUDF), an administrative data set maintained by the Texas Department of State Health Services [14]. The TIPUDF includes approximately 97% of hospital discharges in the state and its characteristics were previously described [15].

We initially extracted data on all patients aged ≥18 years, who were hospitalized between the years 2009 and 2014. We then identified among these hospitalizations those with a diagnosis of cirrhosis and further identified, among the latter, hospitalizations with in-hospital CPR. This latter group formed the primary analytic cohort. A diagnosis of cirrhosis was based on the presence of International Classification of Diseases, Ninth Revision, Clinical Modification codes (ICD-9) 571.2, 571.5, and 571.6 in the principal or secondary diagnosis fields. These codes were used in prior studies of cirrhosis in administrative data [4, 16] and were reported to have a positive predictive value of 90% and negative predictive value of 87% [17]. CPR was identified based on the presence of ICD-9 codes 99.60 or 99.63 [6, 10]. Hospitalizations with a diagnosis of liver transplantation and those with principal diagnoses of cardiac arrest (ICD-9 code 427.5), ventricular fibrillation (ICD-9 code 427.41) or ventricular flutter (ICD-9 code 427.42) were excluded.

In order to identify secular trends not captured through analysis of the primary cohort, we anchored the data to similar-aged hospitalizations during 2009–2014 without a diagnosis of cirrhosis who had in-hospital CPR. In addition, we have examined all hospitalizations aged ≥18 years during the years 2009–2014 with and without a diagnosis of cirrhosis and those who died during hospitalization within each group to estimate the annual rates of in-hospital CPR among all hospitalizations and among hospital decedents.

### Outcomes

The co-primary outcomes were rates of in-hospital CPR among all hospitalizations with and without cirrhosis and rates of short-term survival among hospitalizations with in-hospital CPR. We defined short-term survival as that of hospitalizations without in-hospital death or discharge to hospice.

The secondary outcomes included a) overall and over time rates of in-hospital CPR among all hospitalizations with and without cirrhosis who died in the hospital and b) overall and over time short-term survival among those with and without shockable rhythm, and hospital disposition of those without in-hospital death or discharge to hospice, both among hospitalizations with and without cirrhosis who have undergone in-hospital CPR.

### Study variables

Study variables were selected based on clinical plausibility and prior reports [4, 6, 10]. The abstracted variables included: a) demographics (age, gender, race/ethnicity, and health insurance) b) comorbid conditions, based on the Deyo modification of the Charlson Comorbidity Index [18] c) type of admission (elective vs. non-elective) d) admission during the weekend (vs. during weekdays) e) presence of a shockable rhythm (ICD-9 codes 427.41 or 427.42) [10, 19]; these codes were reported to have positive predictive values between 74% [20] and 100% [21] in administrative data f) number of failing organs [22] g) cirrhosis-related complications (ascites, hepatic encephalopathy, hepatorenal syndrome, hepatocellular cancer, and variceal bleeding) [4] h) sepsis g) hospitals’ teaching status h) hospital disposition and i) year of admission.

### Data analysis

We summarized categorical variables as numbers and percentages, while continuous variables were reported as means (standard deviation [SD]). Group comparisons were performed using chi-square test for categorical variables and t-test or Mann-Whitney test for continuous variables. We used hospitalizations as the unit of analysis because the TIPUDF does not identify individual patients and thus does not capture repeated hospitalizations.

In order to provide further anchoring context to the trends of outcome trajectories between hospitalizations with and without cirrhosis who had in-hospital CPR, we examined their respective temporal trends of the burden of chronic illness and illness severity using the Deyo comorbidity index and the number of failing organs, respectively, as proxy measures.

Temporal trends of the primary and secondary outcomes were examined using weighted least square regression, with calendar year as predictor. Modeled findings are reported as average annual percent change (AAPC) and 95% confidence intervals (95% CI). All temporal analyses were carried out separately for hospitalizations with and without cirrhosis. Comparison of regression slopes was performed using the methods described by Armitage et al [23].

We estimated the risk-adjusted of short-term survival following in-hospital CPR among hospitalizations with and without cirrhosis using empirical Bayesian posterior estimates from multivariate logistic regression models for each (see modeling approach below) and then derived the corresponding annual rates of short-term survival.

A multivariate logistic regression model was fitted to estimate the association between cirrhosis as independent predictor and short-term survival following in-hospital cardiopulmonary resuscitation as dependent variable. Predictors with p < 0.1 on univariate logistic regression were considered for multivariate analysis, following examination for multicollinearity. Candidate predictor covariates were then entered into the model using backward stepwise selection. Results of the multivariate models were expressed as adjusted odds ratios (aOR) and their 95% CI.

The potential predictors of short-term survival following in-hospital cardiopulmonary resuscitation among hospitalizations with cirrhosis were examined using multivariate logistic regression modeling, employing the approach described above for the whole cohort.

### Sensitivity analysis

In order to examine the robustness of our findings and to facilitate comparison to prior studies we re-examined both temporal trends and logistic regression models by substituting the outcome of short-term survival, as defined earlier, with hospital survival, defined as hospitalization without in-hospital death, among hospitalizations with in-hospital CPR, with and without cirrhosis, as well as among the corresponding strata with and without a shockable rhythm and those aged ≥ 65 years. Because the number of independent predictors of short-term survival among hospitalizations with cirrhosis was unexpectedly substantially more limited than among those without cirrhosis, we have repeated the multivariate logistic regression model without the cirrhosis-related complications to examine whether the findings of the original model were driven by the latter complications.

Data analyses were carried out on MedCalc version 17.5.5 (MedCalc Software, Ostend, Belgium). A 2-sided *p* value < 0.05 was considered statistically significant.

## Results

The total number of hospitalizations with and without cirrhosis during the study period was 330,069 and 12,962,435, respectively. There were 19,320 in-hospital deaths among hospitalizations with cirrhosis and 269,978 deaths among those without cirrhosis, corresponding hospital mortality rates of 5.9% and 2.1%, respectively. The details of the annual hospitalization volumes and the corresponding hospital mortality of all hospitalizations with and without cirrhosis are provided in Table A in S1 File. Hospital mortality did not change significantly over time in either group (AAPC [95% CI]: -0.3 [-2.5 to +1.9]; p = 0.7381 for hospitalizations with cirrhosis and -0.1 [-1.6 to +1.3]; p = 0.8047 for hospitalizations without cirrhosis).

### Utilization of in-hospital CPR, patient characteristics, and short-term survival

There were 54,280 hospitalizations with in-hospital CPR during the study period, of which 2,511 were with a diagnosis of cirrhosis and 51,969 without cirrhosis. The characteristics of these hospitalizations are detailed in Table 1. In-hospital CPR was reported among hospitalizations with and without cirrhosis in 7.6 and 4.0 per 1,000 hospitalizations, respectively (p < 0.0001) and in 10.7% vs. 13.4%, respectively, among hospital decedents (p < 0.0001).

**Table 1:** The characteristics of hospitalizations with in-hospital cardiopulmonary resuscitation, with and without cirrhosis.

		Cirrhosis			Non-cirrhosis			*p*
Variables		n = 2,511			n = 51,969			
**Age, years (%)**								<0.0001
18–44		243 (9.7)			5,360 (10.3)			
45–64		1,597 (63.6)			16,732 (32.2)			
≥ 65		671 (26.7)			29,877 (57.5)			
**Gender**^a^	** **							
Female		583 (42.7)			22,668 (46.8)			0.0029
**Race/ethnicity (%)**								<0.0001
White		1,033 (41.1)			26,152 (50.3)			
Hispanic		874 (34.8)			10,662 (20.5)			
Black		394 (15.7)			10,128 (19.5)			
Other		186 (7.4)			4,439 (8.5)			
Missing		24 (0.9)			598 (1.2)			
**Health insurance (%)**								<0.0001
Private		622 (24.8)			13,887 (26.7)			
Medicare		940 (37.4)			28,984 (55.8)			
Medicaid		383 (15.3)			3,513 (6.8)			
Uninsured		500 (19.9)			4,781 (9.2)			
Other		59 (2.3)			717 (1.4)			
Missing		7 (0.3)			87 (0.2)			
**Deyo comorbidity index**^b^		5.6 (2.4)			2.5 (2.2)			<0.0001
**Selected comorbidities**								
Myocardial infarction			306 (12.8)			14,114 (27.2)		<0.0001
Congestive heart failure			726 (28.9)			21,565 (41.5)		<0.0001
Cerebrovascular disease			126 (5.0)			5,957 (11.5)		<0.0001
Diabetes			825 (32.9)			20,099 (38.7)		<0.0001
Malignancy			249 (9.9)			5,819 (11.2)		0.0463
**Elective admission**			270 (10.8)			8,713 (16.8)		<0.0001
**Teaching hospital**			725 (28.9)			13,363 (25.7)		0.0004
**Sepsis**			1,180 (47.0)			19,266 (37.1)		<0.0001
**Number of organ failures**			3.3 (1.4)			2.6 (1.4)		<0.0001
**Shockable rhythm**			185 (7.4)			6,304 (12.1)		<0.0001
**Ascites**			946 (37.7)			NA		
**Hepatic encephalopathy**			501 (20.0)			NA		
**Hepatocellular cancer**			113 (4.5)			NA		
**Hepatorenal syndrome**			236 (9.4)			NA		
**Variceal bleeding**			553 (22.0)			NA		
**Hospital disposition**								
Death			2,061 (82.1)			36,063 (69.4)		<0.0001
Hospice			75 (3.0)			1,705 (3.3)		0.4184
Home			192 (7.2)			6,396 (11.7)		<0.0001
Another hospital			127 (5.0)			5,466 (10.5)		<0.0001
Nursing facility			56 (2.2)			2,339 (4.5)		<0.0001

a Gender was available for 1,364 hospitalizations with cirrhosis and 48,413 hospitalizations without cirrhosis; the percent figures for gender in each column refer to that column’s denominator for gender

b mean (SD)

Percentage figures may not add to 100 due to rounding

The short-term survival following in-hospital CPR was markedly lower among hospitalizations with cirrhosis than among those without cirrhosis (14.9% vs. 27.3%, respectively; p < 0.0001). When stratified by the presence or absence of a shockable rhythm, short-term survival was similar between the two groups among hospitalizations with cirrhosis (14.6% vs. 15.0%, respectively; p = 0.8929), while being markedly higher among those with shockable rhythm among hospitalizations without cirrhosis (36.7% vs. 26.0%, respectively; p < 0.0001).

### Temporal trends among hospitalizations with in-hospital CPR

**Fig 1:**
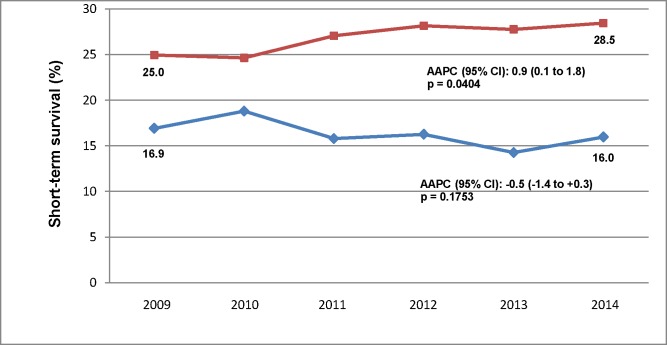
Temporal trends of risk-adjusted short-term survival following in-hospital cardiopulmonary resuscitation. Short-term survival is defined as that of hospitalizations without in-hospital death or discharge to hospice. Hospitalizations with cirrhosis are represented by diamond markers and those without cirrhosis are represented by box markers. AAPC (95% CI) denotes average annual percent change and its 95% confidence intervals. Short-term survival estimates were adjusted for age, race/ethnicity, health insurance, Deyo comorbidity index, myocardial infarction, cerebrovascular disease, diabetes, elective admission, weekend admission, sepsis shockable rhythm, number of organ failures, and year of admission. Hospitalizations with cirrhosis were adjusted in addition for hepatorenal syndrome and hepatocellular cancer.

The temporal patterns of the rates of in-hospital CPR, presence of a shockable rhythm, and related short-term survival among hospitalizations with and without cirrhosis are described in Table 2. The details of the annual volumes of corresponding strata are provided in Table A in S1 File. The temporal trends of risk-adjusted short-term survival following in-hospital CPR among hospitalizations with and without cirrhosis are presented in Fig 1.

**Table 2:** Temporal changes among hospitalizations with in-hospital cardiopulmonary resuscitation, with and without cirrhosis.

				Year					
Category		2009	2010	2011	2012	2013	2014	AAPC (95% CI)^a^	p
**Rate of CPR (all)**^**b**^									
Cirrhosis		7.8	7.0	7.2	8.2	7.5	7.7	0.9 (-3.0 to +4.8)	0.5720
Non-cirrhosis		3.8	3.8	3.9	4.1	4.2	4.3	2.6 (2.0 to 3.1)	0.0002
**CPR among decedents (%)**^**c**^									
Cirrhosis		10.4	9.9	10.7	11.4	10.7	10.8	1.4 (-1.5 to +4.3)	0.2412
Non-cirrhosis		13.0	12.8	13.4	13.7	13.6	13.7	1.3 (0.3 to 2.3)	0.0229
**Shockable rhythm (%)**									
Cirrhosis		9.2	8.3	7.5	6.3	6.9	6.8	-6.4 (-11.8 to -1.0)	0.0309
Non-cirrhosis		11.2	12.0	11.5	12.7	12.1	13.2	2.7 (0.2 to 5.3)	0.0400
**Short-term survival (%)**^**d**^									
**All**									
Cirrhosis		16.4	15.5	14.1	15.9	14.4	13.9	-2.6 (-6.2 to +0.9)	0.1098
Non-cirrhosis		25	27.2	26.5	28.6	28.5	27.9	2.2 (-0.9 to 4.5)	0.0565
**Shockable rhythm**									
Cirrhosis		12.5	21.4	3.4	20.0	9.4	20.6	5.0 (-47.0 to +57.1)	0.8001
Non-cirrhosis		36.4	36.1	34.7	36.9	39.1	36.8	1.0 (-1.5 to +3.6)	0.3208
**Non-shockable rhythm**									
Cirrhosis		16.8	14.9	15.0	15.7	14.6	13.4	-3.0 (-6.4 to -0.2)	0.0432
Non-cirrhosis		23.6	26.0	25.4	27.4	27.0	26.6	2.3 (-0.3 to +4.8)	0.0686

a AAPC: average annual percent change; 95% CI: 95% confidence intervals

b rate of cardiopulmonary resuscitation per 1,000 hospitalizations among all hospitalizations within a specific stratum

c Percent of cardiopulmonary resuscitation among all patients who died in the hospital within a specific stratum

d Short-term survival was defined as that of hospitalizations without in-hospital death or discharge to hospice

The annual rates of in-hospital CPR remained unchanged among hospitalizations with cirrhosis, while rising on average by 2.6%/year among those without cirrhosis. Similarly, the annual rates of in-hospital CPR among hospital decedents remained unchanged among hospitalizations with cirrhosis, while rising slightly, but statistically significantly, among those without cirrhosis.

There was no significant change over time in the crude short-term survival following in-hospital CPR among hospitalizations with or without cirrhosis. However, while the risk-adjusted short-term survival following in-hospital CPR remained unchanged among hospitalizations with cirrhosis, it increased over time among those without cirrhosis (Fig 1). The Deyo comorbidity index and the number of organ failures rose progressively over the study period among hospitalizations with and without cirrhosis who have undergone in-hospital CPR. However, there was no difference between the slopes of either measure between hospitalizations with and without cirrhosis (Table B in S1 File).

The annual rates of discharge to hospice following in-hospital cardiopulmonary resuscitation remained unchanged among hospitalizations with cirrhosis (AAPC = -3.1 [95% CI -12.4 to +18.6]; p = 0.6079), while rising 6.7%/year among those without cirrhosis (95% CI 3.9 to 9.5; p = 0.0026). Transfers to another hospital following in-hospital cardiopulmonary resuscitation did not change over time for hospitalizations with (p = 0.9901) or without cirrhosis (p = 0.9910). Discharges to a nursing facility remained unchanged overtime among hospitalizations with cirrhosis (p = 0.2004), while rising 8.2%/year among those without cirrhosis (95% CI 4.2 to 12.1; p = 0.0045).

### Predictors of short-term survival

The predictors of short-term survival following in-hospital CPR for the whole cohort and among hospitalizations with cirrhosis are described in Tables 3 and 4, respectively.

**Table 3:** Univariate and multivariate logistic regression analysis of predictors of short-term survival among all hospitalizations with in-hospital cardiopulmonary resuscitation.

	Unadjusted odds ratio		Adjusted odds ratio	
Variables	(95% CI)	p	(95% CI)	p
**Age (years)**				
18–44	Reference		Reference	
45–64	0.91 (0.85–0.97)	0.005	0.93 (0.88–0.98)	0.0073
≥65	0.67 (0.63–0.71)	<0.0001	0.71 (0.65–0.77)	<0.0001
**Race/ethnicity**				
White	Reference		Reference	
Hispanic	0.90 (0.86–0.95)	0.0001	0.93 (0.88–0.97)	0.0028
Black	0.87 (0.82–0.91)	<0.0001	0.89 (0.85–0.94)	<0.0001
Other	0.96 (0.90–1.03)	0.3318	NA	
**Health insurance**				
Medicare	Reference		Reference	
Private	1.28 (1.23–1.34)	<0.0001	1.09 (1.04–1.14)	0.0001
Medicaid	1.20 (1.12–1.29)	<0.0001	NA	
No insurance	0.92 (0.86–0.98)	0.0186	0.72 (0.66–0.77)	<0.0001
Other	1.09 (0.93–1.28)	0.2565	NA	
**Cirrhosis**	0.46 (0.41–0.52)	<0.0001	0.55 (0.49–0.62)	<0.0001
**Deyo comorbidity index**	0.94 (0.93–0.95)	<0.0001	0.96 (0.95–0.97)	<0.0001
**Myocardial infarction**	1.17 (1.12–1.22)	<0.0001	1.11 ((1.06–1.16)	<0.0001
**Congestive heart failure**	1.35 (1.30–1.41)	<0.0001	NA	
**Cerebrovascular disease**	1.15 (1.09–1.22)	<0.0001	1.20 (1.13–1.27)	<0.0001
**Diabetes**	1.06 (1.01–1.10)	0.0047	1.54 (1.44–1.66)	<0.0001
**Malignancy**	0.51 (0.47–0.54)	<0.0001	NA	
**Sepsis**	0.61 (0.59–0.64)	<0.0001	0.68 (0.65–0.71)	<0.0001
**Elective admission**	1.46 (1.39–1.53)	<0.0001	1.42 (1.35–1.49)	<0.0001
**Weekend admission**	0.89 (0.85–0.93)	<0.0001	0.94 (0.89–0.98)	0.0111
**Teaching hospital**	0.98 (0.94–1.03)	0.6079	NA	
**Number of organ failures**	0.88 (0.87–0.90)	<0.0001	0.92 (0.91–0.93)	<0.0001
**Shockable rhythm**	1.65 (1.56–1.74)	<0.0001	1.52 (1.43–1.61)	<0.0001
**Year of admission**	1.02 (1.01–1.03)	<0.0001	1.03 (1.02–1.04)	<0.0001

**Table 4:** Univariate and multivariate logistic regression analysis of predictors of short-term survival following in-hospital cardiopulmonary resuscitation among hospitalizations with cirrhosis.

	Unadjusted odds ratio		Adjusted odds ratio	
Variables	(95% CI)	p	(95% CI)	p
**Age (years)**			NA	
18–44	Reference			
45–64	0.99 (0.67–1.46)	0.9789		
≥65	1.17 (0.78–1.77)	0.4358		
**Race/ethnicity**			NA	
White	Reference			
Hispanic	0.80 (0.62–1.03)	0.0851		
Black	0.80 (0.57–1.12)	0.202		
Other	0.89 (0.58–1.38)	0.6331		
**Health insurance**				
Medicare	Reference		Reference	
Private	0.94 (0.71–1.24)	0.6846	NA	
Medicaid	0.95 (0.69–1.32)	0.8009	NA	
No insurance	0.43 (0.30–0.62)	<0.0001	0.47 (0.34–0.67)	<0.0001
Other	0.87 (0.42–1.82)	0.7256	NA	
**Deyo comorbidity index**	1.01 (0.96–1.06)	0.5328	NA	
**Myocardial infarction**	1.42 (1.04–1.94)	0.0234	1.30 (0.95–1.78)	0.0997
**Congestive heart failure**	2.06 (1.64–2.58)	<0.0001	NA	
**Cerebrovascular disease**	1.84 (1.20–2.82)	0.0047	1.83 (1.18–2.82)	0.0066
**Diabetes**	1.18 (0.93–1.50)	0.1586	NA	
**Malignancy**	0.76 (0.50–1.15)	0.2093	NA	
**Sepsis**	0.61 (0.48–0.76)	<0.0001	0.67 (0.53–0.85)	0.0011
**Elective admission**	1.41 (1.02–1.95)	0.0356	1.11 (0.83–1.25)	0.5382
**Weekend admission**	1.02 (0.78–1.32)	0.8754	NA	
**Teaching hospital**	1.01 (0.79–1.28)	0.9285	NA	
**Number of organ failures**	0.85 (0.79–0.92)	0.0001	0.90 (0.83–0.98)	0.0159
**Shockable rhythm**	0.97 (0.63–1.48)	0.8929	NA	
**Ascites**	0.89 (0.71–1.12)	0.3390	NA	
**Hepatic encephalopathy**	0.92 (0.70–1.22)	0.5925	NA	
**Hepatocellular cancer**	0.36 (0.16–0.78)	0.0104	0.35 (0.16–0.76)	0.0086
**Hepatorenal syndrome**	0.41 (0.25–0.68)	0.0007	0.51 (0.30–0.85)	0.0106
**Variceal bleeding**	0.81 (0.62–1.07)	0.1531	NA	
**Year of admission**	0.96 (0.90–1.03)	0.3299	NA	

Cirrhosis was associated with 45% lower odds of short-term survival following in-hospital CPR on adjusted analysis. Among hospitalizations with cirrhosis who had in-hospital CPR, hepatocellular cancer (aOR = 0.35 [95% CI 0.16–0.76]) and lack of health insurance (aOR = 0.47 [95% CI 0.33–0.67]) were associated with the lowest odds of short-term survival. The predictors of short-term survival following in-hospital CPR among hospitalizations without cirrhosis are detailed in Table C in S1 File.

### Sensitivity analysis

When hospital survival following in-hospital CPR was used as the outcome instead of short-term survival, the former remained significantly lower among hospitalizations with cirrhosis than among those without cirrhosis (17.9% vs. 30.6%, respectively; p < 0.0001). The hospital survival following in-hospital CPR among those aged ≥65 years with and without cirrhosis was 20.1% vs. 28.0%, respectively (p < 0.0001). On trend analysis (Table D in S1 File), hospital survival following in-hospital CPR remained unchanged among those with cirrhosis, while the upward trend in short-term survival noted among hospitalizations without cirrhosis became statistically significant for hospital survival. When re-analyzed without cirrhosis-related complications, there were no additional independent predictors of short-term survival and the effect size of the remainder independent predictors did not change significantly (Table E in S1 File).

Using hospital survival as outcome of interest, cirrhosis remained associated independently with worse outcome (OR = 0.55 [95% CI 0.50–0.62]; p < 0.0001) (Table F in S1 File), and among hospitalizations with cirrhosis the statistically significant predictors of adverse outcome remained unchanged (Table G in S1 File).

## Discussion

In this population-based study, the rate of in-hospital CPR was nearly 2-fold higher among hospitalizations with cirrhosis than those without it, though it was applied more selectively among the former. Risk-adjusted short-term survival following in-hospital CPR was markedly lower among hospitalizations with cirrhosis and did not change significantly over time, while improving among hospitalizations without cirrhosis. Lack of health insurance, sepsis, and increasing number of organ failures were associated with lower odds of short-term survival following in-hospital CPR among cirrhosis hospitalizations.

### Relationship to prior studies

The comparative rates of in-hospital CPR between patients with cirrhosis and contemporaneous non-cirrhotic patients were not previously reported, to our knowledge. Our finding of higher rates of in-hospital CPR among cirrhotic patients likely reflects their markedly higher burden of chronic illness and acute severity of illness, associated with the documented higher rate of in-hospital death. On the other hand, in-hospital CPR was used more selectively among cirrhosis hospitalizations, evidenced by its persistently lower use among hospital decedents with cirrhosis, as compared with those without cirrhosis.

An earlier population-based study of Medicare patients aged ≥67 years by Stapleton and colleagues showed markedly lower hospital survival among cirrhotic patients between 1994 and 2005, as compared to those without cirrhosis [6], and a preliminary report described similar findings in all adults during 2000–2009 [7]. Our study is consistent with these observations in a contemporaneous cohort.

Despite the lower hospital survival following in-hospital CRP among cirrhotic patients in the present study, as compared to noncirrhosis patients, survival rates appear to rise in among the former. Thus, hospital survival among those aged ≥65 years has been nearly 2-fold higher in the present study, as compared to that reported by Stapleton et al (20.1% vs. 12.6% [6], respectively), and increased more slowly among all adults from 13% during 2000–2009 [7] to 17.9% in the present study.

There have been no population-level data, to our knowledge, on the epidemiology of shockable vs. nonshockable rhythm among cirrhotic patients undergoing in-hospital CPR, though no difference was found in the rate of a shockable rhythm between patients with and without cirrhosis in a small, single-center cohort [8]. Our finding of markedly lower rates of a shockable rhythm among hospitalizations with cirrhosis who have undergone in-hospital CPR than among noncirrhosis hospitalizations may reflect in part differences in the proximate causes of cardiac arrest and patients’ location at the time. However, administrative data preclude more detailed analysis.

When examined in isolation, our findings of unchanged adjusted short-term survival over time following in-hospital CPR among cirrhosis patients, coupled with their progressive rise in the burden of chronic illness and severity of illness, may be interpreted as reflecting improving care in an increasingly sick population. However, such postulate is not supported by the contemporaneously improving adjusted short-term survival among those without cirrhosis, despite similar upward trajectories of measures of chronic illness and severity of illness. Nevertheless, the severity of comorbidities and organ failures may have diverged over time between patients with and without cirrhosis, exceeding that among the latter, though these data are not available in administrative data sets.

Alternatively, the lack of improvement in short-term survival among cirrhosis hospitalizations, as compared to those without cirrhosis, may have been in part to the progressively decreasing rates of shockable rhythm among the former. However, a key finding of the present study is the similar short-term survival following in-hospital CPR among cirrhosis patients regardless of presence or absence of a shockable rhythm. This finding, not previously reported to our knowledge, contrasts the well-documented prognostic advantage of a shockable rhythm in the general population [10, 11]. The underlying sources of the observed divergent prognostic association of a shockable rhythm between patients with and without cirrhosis are unclear and require further study.

The factors underlying the worse risk-adjusted short-term outcomes following in-hospital CPR among patients with cirrhosis remain incompletely understood. A recent report by Roedl et al documented markedly worse profile of organ failure following CPR among cirrhotic patients, as compared to those without the disease [13], which may have contributed to the lower survival among the former. In addition, as noted earlier, although we controlled our modeling for the burden of chronic illness and the number of failing organs, the severity of neither could be captured. Finally, patients’ location, the proximate precipitant of cardiac arrest, and care during and following cardiac arrest may have differed between cirrhosis and non-cirrhosis hospitalizations. Further studies of the abovementioned data gaps are needed to enhance understanding of the observed outcome patterns in the present study and inform future preventive and intervention strategies.

We found that with the exception of hepatocellular cancer, lack of health insurance was associated with the worst odds of short-term survival among cirrhotic hospitalizations undergoing in-hospital CPR. Lack of health insurance has likely adversely affected patients’ access to primary and specialty care, and thus may have led to worse control of cirrhosis and other comorbidities. In addition, lack of health insurance was shown to be associated with higher mortality among critically ill patients in the general population [24].

Several comorbid conditions, including myocardial infarction, cerebrovascular disease, and diabetes in the whole cohort, and cerebrovascular disease and diabetes and among hospitalizations with cirrhosis were found, unexpectedly, to be associated with increased odds of short-term survival following in-hospital CPR. The factors underlying these findings are unclear. However, each of these comorbidities is associated with increased risk of acute cardiac events and prior studies found that coronary artery disease is associated with increased survival following cardiac arrest [10, 12, 19]. In addition, primary cardiac diagnosis for cardiac arrest was found to be associated with 70% higher odds of survival following in-hospital CPR, as compared to a respiratory diagnosis [25]. It may be hypothesized that the abovementioned comorbidities serve as proxies for a subgroup of patients who are more likely than others to develop in-hospital cardiac arrest due to an acute cardiac event, rather than due to the more common occurrence of cardiac arrest due to progression of another life-limiting condition (e.g., cancer) or due to other acute illness. Thus, patients with these comorbidities may have better odds of survival following in-hospital cardiac arrest *only in comparison* with those without these comorbidities. Additional studies, using more granular data, are needed to examine the factors preceding in-hospital cardiac arrest across the comorbidity and acute illness spectrum to evaluate the validity of our postulate.

A striking finding of the present study was the markedly limited number of factors that were independently associated with short-term survival following in-hospital CPR among hospitalizations with cirrhosis, in contrast to their non-cirrhotic counterparts, and to the markedly broader range of predictors of hospital mortality among cirrhosis hospitalizations in general [4]. Specifically, commonly impactful sociodemographic factors, the burden of other comorbidities or the circumstances of hospital admission (e.g., weekend admission) no longer affected cirrhotic patients’ short-term survival following CPR for in-hospital cardiac arrest. The factors underlying these latter observations, likely substantially driven by peri-arrest pathobiology of cirrhotic patients, warrant further study.

### Implications of study findings

Given the gradual rise of hospital survival over the past decades among patients with cirrhosis undergoing in-hospital CPR, our findings suggest its use has a potential to benefit select patients in this population. Indeed, our study demonstrates that in-hospital CPR continued to be used selectively among cirrhotic hospitalizations, despite the rising hospitalization burden in this population [4]. However, the characteristics of those most likely to benefit have not been systematically examined. In addition, studies are needed to better understand the proximate causes of cardiac arrest among cirrhotic patients and their pathobiology following CPR, in order to inform future preventive and interventional efforts, and to examine the neurological and other functional outcomes among survivors.

Our finding of temporally divergent trajectories of discharge to hospice among hospitalizations with and without cirrhosis who had in-hospital CPR underscores the need for caution when interpreting the longitudinal patterns of hospital mortality-based outcomes in the general population and in comparative population studies, given the dynamic changes in illness complexity and discharge practices.

Last, our findings on the adverse prognostic factors following in-hospital CPR in cirrhotic patients underscore the importance of assuring adequate health insurance, early recognition and timely care of infection and sepsis and, when applicable, interventions to limit evolvement of organ failure.

### Study limitations

Our study has several important limitations, in addition to those noted earlier, related predominantly to its retrospective design and use of administrative data. First, use of administrative data may have led to misclassification of some of the examined hospitalizations, though similar approach was used in other epidemiological studies [4, 6, 10, 16]. Nevertheless, there are limited data on the validity of the ICD-9 procedure codes for CPR in administrative data. A recent registry-based study found considerable underestimation of in-hospital CPR events by these ICD codes, with slightly lower survival than in the reference data [26]. However, there is no evidence for systematic differences in coding for CPR between the general population and patients with cirrhosis. Thus, the low sensitivity of the ICD codes for CPR would not explain the gaps in short-term survival following in-hospital CPR between hospitalizations with and without cirrhosis, nor the divergent temporal trajectories of short-term survival between these groups. Second, although we adjusted for a broad array of patient- and hospital-level covariates, residual confounding cannot be excluded. Last, it is unknown whether our observations reflect those in other states or nationally.

## Conclusions

In-hospital CPR was performed much more commonly among cirrhosis hospitalizations than among those without the disease, though it was used more selectively among the former. Short-term survival following in-hospital CPR remained markedly lower and unchanged among cirrhosis hospitalizations, while progressively rising among their non-cirrhosis counterparts, despite similar rise in illness severity measures over time.

Cirrhosis was associated with substantially lower short term survival following in-hospital CPR on adjusted analyses, while lack of health insurance and sepsis adversely affected outcomes among cirrhosis hospitalizations. Improving access to health insurance and early identification and control of infectious complications may help improve outcomes of patients with cirrhosis who undergo in-hospital CPR.

Additional studies of cirrhosis patients are warranted to gain better understanding of the precipitants of in-hospital cardiac arrest, unique elements of their pathobiology following return of spontaneous circulation, and functional outcomes beyond short-term survival, in order to identify those more likely to benefit from CPR and to inform preventive and interventional strategies to improve outcomes.

## Supporting information

S1 File Supporting information file. (DOCX)
