# Supplementary material for: In-hospital cardiopulmonary resuscitation of patients with cirrhosis: A population-based analysis
Source: PLoS One. 2019 Sep 30;14(9):e0222873. doi: 10.1371/journal.pone.0222873 (PMC6768467; doi:10.1371/journal.pone.0222873)
Supplement: S1 File — (DOCX) [file pone.0222873.s001.docx]

In-Hospital Cardiopulmonary Resuscitation of Patients with Cirrhosis: a Population-Based Analysis

Lavi Oud, MD

Supplementary Data

**Appendix A: Materials and Methods**

**The details of Materials and Methods that were not included in the main manuscript are italicized.**

This was a retrospective, population-based cohort study. Because we used a publicly available, de-identified data set, the study was determined to be exempt from formal review by the Texas Tech Health Sciences Center’s Institutional Review Board.

*Data sources and study population*

We used the Texas Inpatient Public Use Data File (TIPUDF), an administrative data set maintained by the Texas Department of State Health Services [1]. The TIPUDF includes approximately 97% of hospital discharges in the state and its characteristics were previously described [2].

We extracted initially data on all patients aged ≥18 years, who were hospitalized between the years 2009 and 2014. We then identified among these hospitalizations those with a diagnosis of cirrhosis and subsequently identified, among the latter, hospitalizations with in-hospital cardiopulmonary resuscitation. This latter group formed the primary analytic cohort. A diagnosis of cirrhosis was based on the presence of International Classification of Diseases*,* Ninth Revision*,* Clinical Modification codes (ICD-9) 571.2, 571.5, and 571.6 in the principal or secondary diagnosis fields. These codes were used in prior studies of cirrhosis in administrative data [3, 4] and were reported to have a positive predictive value of 90% and negative predictive value of 87% [5]. Cardiopulmonary resuscitation was identified based on the presence of ICD-9 codes 99.60 or 99.63. Similar approach was used in prior studies of in-hospital cardiopulmonary resuscitation in administrative data [6, 7]. Hospitalizations with a diagnosis of liver transplantation and those with a principal diagnosis of cardiac arrest (ICD-9 code 427.5), ventricular fibrillation (ICD-9 code 427.51) or ventricular flutter (ICD-9 code 427.52) were excluded.

In order to identify secular trends not captured through analysis of the primary cohort, we anchored the data to similar-aged hospitalizations during 2009-2014 without a diagnosis of cirrhosis who had in-hospital cardiopulmonary resuscitation. In addition, we have examined all hospitalizations aged ≥18 years during the years 2009-2014 with and without a diagnosis of cirrhosis and those who died during hospitalization within each group to estimate the annual rates of in-hospital cardiopulmonary resuscitation among all hospitalizations and among hospital decedents.

The institutional Review Board at the Texas Tech University Health Sciences Center deemed this study exempt from review due to use of de-identified data.

*Outcomes*

The co-primary outcomes were rates of in-hospital cardiopulmonary resuscitation among all hospitalizations with and without cirrhosis and rates of short-term survival among hospitalizations with in-hospital cardiopulmonary resuscitation. *Both outcomes were examined overall and over time. Because discharge to hospice is increasingly common among hospitalized patients [3, 8] and specifically, following in-hospital CPR [9] there is a potential for overestimation of short-term survival of the inpatient population when examined over time, if discharges to hospice are not considered [8]. Moreover, interpretation of comparative secular trends of short-term survival of examined select inpatient populations may be compromised when discharge to hospice occurs at different rates overall and over time between groups. We thus defined short-term survival as that of hospitalizations without in-hospital death or discharge to hospice.*

The secondary outcomes among hospitalizations with in-hospital cardiopulmonary resuscitation with and without cirrhosis included overall and over time rates of in-hospital cardiopulmonary resuscitation among those who died in the hospital, short-term survival among those with and without shockable rhythm, and hospital dispositions of those without in-hospital death or discharge to hospice.

*Study variables*

Study variables were selected based on clinical plausibility and prior reports (refs). The abstracted variables included: a) demographics (age, gender, race/ethnicity, and health insurance) b) comorbid conditions, based primarily on the Deyo modification of the Charlson Comorbidity Index [10] c) type of admission (elective vs. non-elective) d) admission during the weekend (vs. during weekdays) e) presence of a shockable rhythm (ICD-9 codes 427.51 or 427.52) [11, 12] f) the number of organ failures, *based on the approach reported by Martin and colleagues [13], used as a proxy for severity of illness [14]* g) hospitals’ teaching status h) hospital disposition and i) year of admission. *In addition, we abstracted cirrhosis-related complications, including ascites, hepatic encephalopathy, hepatorenal syndrome, hepatocellular cancer, and variceal, using the algorithmic approach described by Schmidt and colleagues [3].*

*Last, we included sepsis among examined covariates because cirrhosis is associated with markedly higher risk of infection and resultant sepsis events than the general population [15], with sepsis reported to increase, in turn, the risk of acute decompensation events among cirrhotic patients [16, 17], and because sepsis has been estimated to be present in 1 out of 2 to 3 hospital deaths in the United States [18] and thus can be expected to be a major modulator of short-term survival among patients undergoing in-hospital cardiopulmonary resuscitation. Because the optimal approach to identify sepsis in administrative data is unknown, we used a combination of the taxonomy described by Angus et al [19] and “explicit” ICD-9 codes for severe sepsis (995.92) and septic shock (785.52), as previously described [20].*

*Data analysis*

We summarized categorical variables as numbers and percentages, while continuous variables were reported as means (standard deviation [SD]) or medians (interquartile range [IQR]), as appropriate. Group comparisons were performed using chi-square test for categorical variables and t-test or Mann-Whitney test for continuous variables, as appropriate. We used hospitalizations as the unit of analysis because the TIPUDF does not identify individual patients and thus does not capture repeated hospitalizations.

In order to provide further anchoring context to the comparative trajectories of the observed secular trends between hospitalizations with and without cirrhosis who had in-hospital cardiopulmonary resuscitation, we examined their respective temporal trends of the burden of chronic illness and illness severity using the Deyo comorbidity index and the number of failing organs, respectively, as proxy measures.

Temporal trends of the primary and secondary outcomes were examined using weighted least square regression, with calendar year as predictor. Modeled findings are reported as average annual percent change (AAPC) and 95% confidence intervals (95% CI). All temporal analyses were carried out separately for hospitalizations with and without cirrhosis. Comparison of regression slopes was performed using the methods described by Armitage et al [21].

We estimated the risk-adjusted of short-term survival following in-hospital cardiopulmonary resuscitation among hospitalizations with and without cirrhosis using empirical Bayesian posterior estimates from multivariate logistic regression models for each (see modeling approach below) and then derived the corresponding annual rates of short-term survival.

A multivariate logistic regression model was fitted to estimate the association between cirrhosis as independent predictor and short-term survival following in-hospital cardiopulmonary resuscitation as dependent variable. Predictors with p < 0.1 on univariate logistic regression were considered for multivariate analysis, following examination for multicollinearity. Candidate predictor covariates were then entered into the model using backward stepwise selection *and included age, race/ethnicity, health insurance, type of admission (elective vs. non-elective), weekend (vs. weekday) admission, diagnosis of cirrhosis, Deyo comorbidity index (adjusted by excluding liver disease to avoid duplicate analysis), myocardial infarction, cerebrovascular disease, diabetes, sepsis, number of organ failures, shockable (vs. non-shockable) rhythm, and the year of admission.*

The potential predictors of short-term survival following in-hospital cardiopulmonary resuscitation among hospitalizations with cirrhosis were examined using multivariate logistic regression modeling, employing the approach described above for the whole cohort. *The covariates entered into the model included health insurance, type of admission (elective vs. non-elective), myocardial infarction, cerebrovascular disease, sepsis, the number of organ failures, hepatorenal syndrome, and hepatocellular cancer.*

*The state of Texas suppresses gender code data of hospitalizations with a diagnosis of infection with the human immunodeficiency virus (HIV), ethanol abuse or drug abuse. Because gender data was suppressed in 46% of cirrhosis hospitalizations who had in-hospital cardiopulmonary resuscitation, thus rendering gender-related regression findings non-representative for this group, gender was excluded from regression models on the prognostic impact of cirrhosis and those exploring the potential prognostic factors among hospitalizations with cirrhosis.*

*Sensitivity analysis*

In order to examine the robustness of our findings and to facilitate comparison to prior studies we re-examined both temporal trends and logistic regression models by substituting the outcome of short-term survival, as defined earlier, with hospital survival, defined as hospitalization without in-hospital death, among hospitalizations with in-hospital cardiopulmonary resuscitation, with and without cirrhosis, as well the corresponding strata with and without a shockable rhythm. Because the number of independent predictors of short-term survival among hospitalizations with cirrhosis was substantially more limited than among those without cirrhosis, we have repeated the multivariate logistic regression model without the cirrhosis-related complications to examine whether the findings of the original model were driven by the latter complications.

Data analyses were carried out on MedCalc version 17.5.5 (MedCalc Software, Ostend, Belgium). A 2-sided *p* value < 0.05 was considered statistically significant.

| **Table A. The annual volume of hospitalizations with and without cirrhosis, overall and for specific strata** | | | | | | |  |
| --- | --- | --- | --- | --- | --- | --- | --- |
|  |  |  |  |  | |  |  |
|  |  |  |  | **Year** | |  |  |
| **Category** |  | **2009** | **2010** | **2011** | **2012** | **2013** | **2014** |
| **Number of hospitalizations (all)^a^** | |  |  |  |  |  |  |
| Cirrhosis |  | 44,447 | 47,731 | 53,954 | 58,096 | 61,615 | 64,226 |
| Non-cirrhosis | | 2,158,112 | 2,170,819 | 2,169,599 | 2,181,817 | 2,134,414 | 2,147,674 |
| **Decedents among hospitalizations (all)^b,c^** | |  |  |  |  |  |  |
| Cirrhosis |  | 2,700 (6.1) | 2,811 (5.9) | 3,032 (5.6) | 3,357 (5.8) | 3,582 (5.8) | 3,838 (6.0) |
| Non-cirrhosis | | 45,178 (2.1) | 45,318 (2.1) | 44,811 (2.1) | 44,293 (2.0) | 44,645 (2.1) | 45,553 (2.1) |
| **Hospitalizations with in-hospital CPR** | |  |  |  |  |  |  |
| Cirrhosis |  | 348 | 336 | 389 | 477 | 464 | 497 |
| Non-cirrhosis | | 8,151 | 8,339 | 8,485 | 8,908 | 8,945 | 9,141 |
| **Shockable rhythm among hospitalizations** | |  |  |  |  |  |  |
| **with CPR** |  |  |  |  |  |  |  |
| Cirrhosis |  | 32 | 28 | 29 | 30 | 32 | 34 |
| Non-cirrhosis | | 910 | 999 | 978 | 1,132 | 1,079 | 1,206 |
| **Short-term survival among CPR hospitalizations^d^** | | |  |  |  |  |  |
| **All** |  |  |  |  |  |  |  |
| Cirrhosis | | 57 | 52 | 55 | 76 | 67 | 69 |
| Non-cirrhosis | | 2,040 | 2,266 | 2,245 | 2,550 | 2,549 | 2,551 |
| **Shockable rhythm** | |  |  |  |  |  |  |
| Cirrhosis | | 4 | 6 | 1 | 6 | 3 | 7 |
| Non-cirrhosis | | 331 | 361 | 339 | 418 | 422 | 444 |
| **Non-shockable rhythm** | |  |  |  |  |  |  |
| Cirrhosis | | 53 | 46 | 54 | 70 | 63 | 62 |
| Non-cirrhosis | | 1,709 | 1,905 | 1,906 | 2,132 | 2,127 | 2,107 |
| a All hospitalizations with and without cirrhosis | |  |  |  |  |  |  |
| b Hospitalizations ending with in-hospital death among all hospitalizations with and without cirrhosis | | | | | |  |  |
| c percent of hospitalizations ending with in-hospital death out of all hospitalizations with and without cirrhosis | | | | | | |  |
| d Short-term survival was defined as that of hospitalizations without in-hospital death or discharge to hospice | | | | | | |  |

| **Table B. Linear regression of the annual changes in the Dyeo comorbidity index and the number of organ failures among** | | | | |
| --- | --- | --- | --- | --- |
| **hospitalizations with and without cirrhosis, who have undergone in-hospital cardiopulmonary resuscitation** | | | |  |
|  |  |  |  |  |
| **Category** | **Cirrhosis^a^** | **Non-cirrhosis^a^** | **Difference in regression slopes^b^** |  |
| Deyo comorbidity score | 0.052 (0.007); p < 00001 | 0.069 (0.028); p = 0.0149 | 0.017 (0.027); p = 0.5135 |  |
| Number of organ failures | 0.074 (0.003); p < 0.0001 | 0.071 (0.017); p < 0.0001 | 0.002 (0.016); p = 0.8671 |  |
| a the entries represent regression coefficients (standard errors) and respective p values for each regression model | | | |  |
| b the entries represent comparison of differences in regression slopes between hospitalizations with and without cirrhosis | | | |  |

| \| \| **Table C. Univariate and multivariate logistic regression analysis of predictors of short-term survival** \| \| \| \|  \| \| --- \| --- \| --- \| --- \| --- \| \| **following in-hospital cardiopulmonary resuscitation among hospitalizations without cirrhosis** \| \| \| \|  \| \|  \|  \|  \|  \|  \| \|  \| **Unadjusted odds ratio** \|  \| **Adjusted odds ratio** \|  \| \| **Variables** \| **(95% CI)** \| **p** \| **(95% CI)** \| **p** \| \| **Age (years)** \|  \|  \|  \|  \| \| 18-44 \| Reference \|  \| Reference \|  \| \| 45-64 \| 0.94 (0.88-1.01) \| 0.08009 \| 0.92 (0.87-0.98) \| 0.0351 \| \| ≥65 \| 0.65 (0.61-0.69) \| <0.0001 \| 0.61 (0.56-0.66) \| <0.0001 \| \| **Female** \| 1.04 (1.01-1.08) \| 0.0257 \| 1.10 (1.05-1.14) \| <0.0001 \| \| **Race/ethnicity** \|  \|  \|  \|  \| \| White \| Reference \|  \| Reference \|  \| \| Hispanic \| 0.93 (0.88-0.98) \| 0.008 \| 0.93( 0.89-0.98) \| 0.0146 \| \| Black \| 0.87 (0.82-0.91) \| <0.0001 \| 0.85 (0.81-0.90) \| <0.0001 \| \| Other \| 0.96 (0.90-1.04) \| 0.3853 \| NA \|  \| \| **Health insurance** \|  \|  \|  \|  \| \| Medicare \| Reference \|  \| Reference \|  \| \| Private \| 1.30 (1.25-1.36) \| <0.0001 \| 1.07 (1.01-1.12) \| 0.0061 \| \| Medicaid \| 1.27 (1.17-1.37) \| <0.0001 \| 1.07 (0.98-1.18) \| 0.0918 \| \| No insurance \| 0.99 (0.92-1.06) \| 0.8101 \| 0.47 (0.33-0.67) \| <0.0001 \| \| Other \| 1.13 (0.96-1.33) \| 0.1301 \| 0.82 (0.69-0.99) \| 0.0392 \| \| **Deyo comorbidity score** \| 0.95 (0.94-0.96) \| <0.0001 \| 0.97 (0.97-0.98) \| <0.0001 \| \| **Myocardial infarction** \| 1.14 (1.09-1.19) \| <0.0001 \| 1.07 (1.02-1.12) \| 0.0026 \| \| **Congestive heart failure** \| 1.32 (1.27-1.37) \| <0.0001 \| NA \|  \| \| **Cerebrovascular disease** \| 1.12 (1.06-1.19) \| 0.0001 \| 1.27 (1.19-1.35) \| <0.0001 \| \| **Diabetes** \| 1.47 (1.37-1.57) \| <0.0001 \| 1.51 (1.40-1.61) \| <0.0001 \| \| **Malignancy** \| 0.50 (0.46-0.54) \| <0.0001 \| NA \|  \| \| **Sepsis** \| 0.62 (0.59-0.65) \| <0.0001 \| 0.71 (0.68-0.74) \| <0.0001 \| \| **Elective admission** \| 1.44 (1.37-1.51) \| <0.0001 \| 1.38 (1.31-1.46) \| <0.0001 \| \| **Weekend admission** \| 0.89 (0.85-0.93) \| <0.0001 \| 0.95 (0.90-0.99) \| 0.0461 \| \| **Teaching hospital** \| 0.99 (0.94-1.03) \| 0.743 \| NA \|  \| \| **Number of organ failures** \| 0.89 (0.88-0.91) \| <0.0001 \| 0.97 (0.95-0.98) \| 0.0002 \| \| **Shockable rhythm** \| 1.64 (1.56-1.74) \| <0.0001 \| 1.55 (1.46-1.65) \| <0.0001 \| \| **Year of admission** \| 1.02 (1.01-1.04) \| <0.0001 \| 1.04 (1.03-1.05) \| <0.0001 \|   **Table D. Hospital survival following in-hospital cardiopulmonary resuscitation** \| \| \| \| \| \| \| \|  \|  \| \| --- \| --- \| --- \| --- \| --- \| --- \| --- \| --- \| --- \| --- \| --- \| --- \| --- \| --- \| --- \| --- \| --- \| --- \| --- \| --- \| --- \| --- \| --- \| --- \| --- \| --- \| --- \| --- \| --- \| --- \| --- \| --- \| --- \| --- \| --- \| --- \| --- \| --- \| --- \| --- \| --- \| --- \| --- \| --- \| --- \| --- \| --- \| --- \| --- \| --- \| --- \| --- \| --- \| --- \| --- \| --- \| --- \| --- \| --- \| --- \| --- \| --- \| --- \| --- \| --- \| --- \| --- \| --- \| --- \| --- \| --- \| --- \| --- \| --- \| --- \| --- \| --- \| --- \| --- \| --- \| --- \| --- \| --- \| --- \| --- \| --- \| --- \| --- \| --- \| --- \| --- \| --- \| --- \| --- \| --- \| --- \| --- \| --- \| --- \| --- \| --- \| --- \| --- \| --- \| --- \| --- \| --- \| --- \| --- \| --- \| --- \| --- \| --- \| --- \| --- \| --- \| --- \| --- \| --- \| --- \| --- \| --- \| --- \| --- \| --- \| --- \| --- \| --- \| --- \| --- \| --- \| --- \| --- \| --- \| --- \| --- \| --- \| --- \| --- \| --- \| --- \| --- \| --- \| --- \| --- \| --- \| --- \| --- \| --- \| --- \| --- \| --- \| --- \| --- \| --- \| --- \| --- \| --- \| --- \| --- \| --- \| --- \| --- \| --- \| --- \| --- \| --- \| --- \| --- \| --- \| --- \| --- \| --- \| --- \| --- \| --- \| --- \| --- \| --- \| --- \| \|  \|  \|  \|  \| **Year** \|  \|  \|  \|  \|  \| \| **Category** \|  \| **2009** \| **2010** \| **2011** \| **2012** \| **2013** \| **2014** \| **AAPC (95% CI)^a^** \| **p** \| \| **Hospital survival (%)** \| \|  \|  \|  \|  \|  \|  \|  \|  \| \| **All** \|  \|  \|  \|  \|  \|  \|  \|  \|  \| \| Cirrhosis \| \| 19.5 \| 17.6 \| 16.7 \| 19.9 \| 17.3 \| 16.9 \| -1.4 (-6.3 to +3.5) \| 0.4801 \| \| Non-cirrhosis \| \| 27.8 \| 30.2 \| 29.4 \| 32 \| 32.2 \| 31.8 \| 2.7 (0.6 to 4.8) \| 0.0245 \| \| **Shockable rhythm** \| \|  \|  \|  \|  \|  \|  \|  \|  \| \| Cirrhosis \| \| 15.6 \| 21.4 \| 6.9 \| 20.0 \| 12.5 \| 26.5 \| 6.0 (-28.7 to +40.7) \| 0.6569 \| \| Non-cirrhosis \| \| 40.1 \| 39.1 \| 37.8 \| 40.0 \| 42.5 \| 40 \| 0.8 (-1.8 to +3.4) \| 0.4194 \| \| **Non-shockable rhythm** \| \| \|  \|  \|  \|  \|  \|  \|  \| \| Cirrhosis \| \| 19.9 \| 17.2 \| 17.5 \| 19.9 \| 17.4 \| 16.2 \| -2.5 (-7.7 to +2.7) \| 0.2515 \| \| Non-cirrhosis \| \| 26.3 \| 29.0 \| 28.3 \| 30.8 \| 30.7 \| 30.5 \| 2.9 (0.6 to 5.2) \| 0.0263 \|  \| **Table E. Univariate and multivariate logistic regression analysis of predictors of short-term survival** \| \| \| \| \| \| \| --- \| --- \| --- \| --- \| --- \| --- \| \| **following in-hospital cardiopulmonary resuscitation among hospitalizations with cirrhosis, without** \| \| \| \| \| \| \| **cirrhosis-related complications** \| \|  \|  \|  \|  \| \|  \|  \|  \|  \|  \|  \| \|  \| **Unadjusted odds ratio** \|  \| **Adjusted odds ratio** \|  \|  \| \| **Variables** \| **(95% CI)** \| **p** \| **(95% CI)** \| **p** \|  \| \| **Age (years)** \|  \|  \| NA \|  \|  \| \| 18-44 \| Reference \|  \|  \|  \|  \| \| 45-64 \| 0.99 (0.67-1.46) \| 0.9789 \|  \|  \|  \| \| ≥65 \| 1.17 (0.78-1.77) \| 0.4358 \|  \|  \|  \| \| **Race/ethnicity** \|  \|  \| NA \|  \|  \| \| White \| Reference \|  \|  \|  \|  \| \| Hispanic \| 0.80 (0.62-1.03) \| 0.0851 \|  \|  \|  \| \| Black \| 0.80 (0.57-1.12) \| 0.202 \|  \|  \|  \| \| Other \| 0.89 (0.58-1.38) \| 0.6331 \|  \|  \|  \| \| **Health insurance** \|  \|  \|  \|  \|  \| \| Medicare \| Reference \|  \| Reference \|  \|  \| \| Private \| 0.94 (0.71-1.24) \| 0.6846 \| NA \|  \|  \| \| Medicaid \| 0.95 (0.69-1.32) \| 0.8009 \| NA \|  \|  \| \| No insurance \| 0.43 (0.30-0.62) \| <0.0001 \| 0.47 (0.33-0.67) \| <0.0001 \|  \| \| Other \| 0.87 (0.42-1.82) \| 0.7256 \| NA \|  \|  \| \| **Deyo comorbidity score** \| 1.01 (0.96-1.06) \| 0.5328 \| NA \|  \|  \| \| **Myocardial infarction** \| 1.42 (1.04-1.94) \| 0.0234 \| 1.36 (0.99-1.87) \| 0.0505 \|  \| \| **Congestive heart failure** \| 2.06 (1.64-2.58) \| <0.0001 \| NA \|  \|  \| \| **Cerebrovascular disease** \| 1.84 (1.20-2.82) \| 0.0047 \| 1.84 (1.19-2.84) \| 0.0058 \|  \| \| **Diabetes** \| 1.18 (0.93-1.50) \| 0.1586 \| NA \|  \|  \| \| **Malignancy** \| 0.76 (0.50-1.15) \| 0.2093 \| NA \|  \|  \| \| **Sepsis** \| 0.61 (0.48-0.76) \| <0.0001 \| 0.66 (0.52-0.84) \| 0.0007 \|  \| \| **Elective admission** \| 1.41 (1.02-1.95) \| 0.0356 \| 1.18 (0.88-1.33) \| 0.4173 \|  \| \| **Weekend admission** \| 1.02 (0.78-1.32) \| 0.8754 \| NA \|  \|  \| \| **Teaching hospital** \| 1.01 (0.79-1.28) \| 0.9285 \| NA \|  \|  \| \| **Number of organ failures** \| 0.85 (0.79-0.92) \| 0.0001 \| 0.89 (0.82-0.97) \| 0.0086 \|  \| \| **Shockable rhythm** \| 0.97 (0.63-1.48) \| 0.8929 \| NA \|  \|  \| \| **Year of admission** \| 0.96 (0.90-1.03) \| 0.3299 \| NA \|  \|  \| | | | | | | | |  |  |
| --- | --- | --- | --- | --- | --- | --- | --- | --- | --- | --- | --- | --- | --- | --- | --- | --- | --- | --- | --- | --- | --- | --- | --- | --- | --- | --- | --- | --- | --- | --- | --- | --- | --- | --- | --- | --- | --- | --- | --- | --- | --- | --- | --- | --- | --- | --- | --- | --- | --- | --- | --- | --- | --- | --- | --- | --- | --- | --- | --- | --- | --- | --- | --- | --- | --- | --- | --- | --- | --- | --- | --- | --- | --- | --- | --- | --- | --- | --- | --- | --- | --- | --- | --- | --- | --- | --- | --- | --- | --- | --- | --- | --- | --- | --- | --- | --- | --- | --- | --- | --- | --- | --- | --- | --- | --- | --- | --- | --- | --- | --- | --- | --- | --- | --- | --- | --- | --- | --- | --- | --- | --- | --- | --- | --- | --- | --- | --- | --- | --- | --- | --- | --- | --- | --- | --- | --- | --- | --- | --- | --- | --- | --- | --- | --- | --- | --- | --- | --- | --- | --- | --- | --- | --- | --- | --- | --- | --- | --- | --- | --- | --- | --- | --- | --- | --- | --- | --- | --- | --- | --- | --- | --- | --- | --- | --- | --- | --- | --- | --- | --- | --- | --- | --- | --- | --- | --- | --- | --- | --- | --- | --- | --- | --- | --- | --- | --- | --- | --- | --- | --- | --- | --- | --- | --- | --- | --- | --- | --- | --- | --- | --- | --- | --- | --- | --- | --- | --- | --- | --- | --- | --- | --- | --- | --- | --- | --- | --- | --- | --- | --- | --- | --- | --- | --- | --- | --- | --- | --- | --- | --- | --- | --- | --- | --- | --- | --- | --- | --- | --- | --- | --- | --- | --- | --- | --- | --- | --- | --- | --- | --- | --- | --- | --- | --- | --- | --- | --- | --- | --- | --- | --- | --- | --- | --- | --- | --- | --- | --- | --- | --- | --- | --- | --- | --- | --- | --- | --- | --- | --- | --- | --- | --- | --- | --- | --- | --- | --- | --- | --- | --- | --- | --- | --- | --- | --- | --- | --- | --- | --- | --- | --- | --- | --- | --- | --- | --- | --- | --- | --- | --- | --- | --- | --- | --- | --- | --- | --- | --- | --- | --- | --- | --- | --- | --- | --- | --- | --- | --- | --- | --- | --- | --- | --- | --- | --- | --- | --- | --- | --- | --- | --- | --- | --- | --- | --- | --- | --- | --- | --- | --- | --- | --- | --- | --- | --- | --- | --- | --- | --- | --- | --- | --- | --- | --- | --- | --- | --- | --- | --- | --- | --- | --- | --- | --- | --- | --- | --- | --- | --- | --- | --- | --- | --- | --- | --- | --- | --- | --- | --- | --- | --- | --- | --- | --- | --- | --- | --- | --- | --- | --- | --- | --- | --- | --- | --- | --- | --- | --- | --- | --- | --- | --- | --- | --- | --- | --- | --- | --- | --- | --- | --- | --- | --- | --- | --- | --- | --- | --- | --- | --- | --- | --- | --- | --- | --- | --- | --- | --- | --- | --- | --- | --- | --- | --- | --- | --- | --- | --- | --- | --- | --- | --- | --- | --- | --- | --- | --- | --- | --- | --- | --- | --- | --- | --- | --- | --- | --- | --- | --- | --- | --- | --- | --- | --- | --- | --- | --- | --- | --- | --- | --- | --- | --- | --- | --- | --- | --- | --- | --- | --- | --- | --- | --- | --- | --- | --- | --- | --- | --- | --- | --- | --- | --- |
|  |  |  |  |  |  |  |  |  |  |
| \| **Table F. Univariate and multivariate logistic regression analysis of predictors of hospital survival** \| \| \| \|  \| \| --- \| --- \| --- \| --- \| --- \| \| **among all hospitalizations with in-hospital cardiopulmonary resuscitation** \| \| \| \|  \| \|  \|  \|  \|  \|  \| \|  \| **Unadjusted odds ratio** \|  \| **Adjusted odds ratio** \|  \| \| **Variables** \| **(95% CI)** \| **p** \| **(95% CI)** \| **p** \| \| **Age (years)** \|  \|  \|  \|  \| \| 18-44 \| Reference \|  \| Reference \|  \| \| 45-64 \| 0.96 (0.90-1.03) \| 0.3031 \| NA \|  \| \| ≥65 \| 0.77 (0.72-0.81) \| <0.0001 \| 0.81 (0.77-0.85) \| <0.0001 \| \| **Race/ethnicity** \|  \|  \|  \|  \| \| White \| Reference \|  \| Reference \|  \| \| Hispanic \| 0.89 (0.85-0.93) \| <0.0001 \| 0.90 (0.86-0.95) \| 0.0001 \| \| Black \| 0.84 (0.80-0.89) \| <0.0001 \| 0.85 (0.81-0.90) \| <0.0001 \| \| Other \| 0.93 (0.87-1.01) \| 0.0624 \| 0.93 (0.86-0.99) \| 0.0413 \| \| **Health insurance** \|  \|  \|  \|  \| \| Medicare \| Reference \|  \| Reference \|  \| \| Private \| 1.21 (1.16-1.26) \| <0.0001 \| 1.05 (1.01-1.10) \| 0.0194 \| \| Medicaid \| 1.16 (1.08-1.25) \| <0.0001 \| NA \|  \| \| No insurance \| 0.84 (0.79-0.90) \| <0.0001 \| 0.70 (0.65-0.75) \| <0.0001 \| \| Other \| 0.97 (0.83-1.14) \| 0.7416 \| NA \|  \| \| **Cirrhosis** \| 0.49 (0.44-0.54) \| <0.0001 \| 0.55 (0.50-0.62) \| <0.0001 \| \| **Deyo comorbidity score** \| 0.96 (0.95-0.97) \| <0.0001 \| 0.98 (0.97-0.99) \| <0.0001 \| \| **Myocardial infarction** \| 1.15 (1.10-1.20) \| <0.0001 \| 1.08 ((1.03-1.12) \| 0.0004 \| \| **Congestive heart failure** \| 1.39 (1.34-1.45) \| <0.0001 \| NA \|  \| \| **Cerebrovascular disease** \| 1.22 (1.15-1.29) \| <0.0001 \| 1.25 (1.18-1.33) \| <0.0001 \| \| **Diabetes** \| 1.04 (1.01-1.08) \| 0.0202 \| 1.52 (1.42-1.62) \| <0.0001 \| \| **Malignancy** \| 0.58 (0.54-0.62) \| <0.0001 \| NA \|  \| \| **Sepsis** \| 0.66 (0.63-0.68) \| <0.0001 \| 0.70 (0.68-0.73) \| <0.0001 \| \| **Elective admission** \| 1.39 (1.32-1.46) \| <0.0001 \| 1.35 (1.29-1.42) \| <0.0001 \| \| **Weekend admission** \| 0.90 (0.86-0.94) \| <0.0001 \| 0.94 (0.90-0.99) \| 0.0244 \| \| **Teaching hospital** \| 0.97 (0.93-1.01) \| 0.1965 \| NA \|  \| \| **Number of organ failures** \| 0.92 (0.91-0.93) \| <0.0001 \| 0.96 (0.94-0.97) \| <0.0001 \| \| **Shockable rhythm** \| 1.60 (1.52-1.69) \| <0.0001 \| 1.50 (1.42-1.58) \| <0.0001 \| \| **Year of admission** \| 1.03 (1.02-1.04) \| <0.0001 \| 1.04 (1.03-1.05) \| <0.0001 \|  \| **Table G. Univariate and multivariate logistic regression analysis of predictors of hospital survival** \| \| \| \|  \| \| --- \| --- \| --- \| --- \| --- \| \| **following in-hospital cardiopulmonary resuscitation among hospitalizations with cirrhosis** \| \| \| \|  \| \|  \|  \|  \|  \|  \| \|  \| **Unadjusted odds ratio** \|  \| **Adjusted odds ratio** \|  \| \| **Variables** \| **(95% CI)** \| **p** \| **(95% CI)** \| **p** \| \| **Age (years)** \|  \|  \| NA \|  \| \| 18-44 \| Reference \|  \|  \|  \| \| 45-64 \| 1.13 (0.78-1.63) \| 0.5106 \|  \|  \| \| ≥65 \| 1.35 (0.91-2.01) \| 0.1275 \|  \|  \| \| **Race/ethnicity** \|  \|  \|  \|  \| \| White \| Reference \|  \|  \|  \| \| Hispanic \| 0.72 (0.56-0.91) \| 0.0069 \| 0.75 (0.60-0.94) \| 0.0159 \| \| Black \| 0.86 (0.64-1.16) \| 0.3471 \| NA \|  \| \| Other \| 0.75 (0.49-1.15) \| 0.1956 \| NA \|  \| \| **Health insurance** \|  \|  \|  \|  \| \| Medicare \| Reference \|  \| Reference \|  \| \| Private \| 1.01 (0.79-1.31) \| 0.8807 \| NA \|  \| \| Medicaid \| 0.95 (0.70-1.24) \| 0.7455 \| NA \|  \| \| No insurance \| 0.42 (0.30-0.59) \| <0.0001 \| 0.45 (0.33-0.63) \| <0.0001 \| \| Other \| 0.91 (0.46-1.78) \| 0.7855 \| NA \|  \| \| **Deyo comorbidity score** \| 1.02 (0.98-1.06) \| 0.2282 \| NA \|  \| \| **Myocardial infarction** \| 1.48 (1.11-1.98) \| 0.0066 \| 1.40 (1.04-1.88) \| 0.0232 \| \| **Congestive heart failure** \| 2.00 (1.62-2.48) \| <0.0001 \| NA \|  \| \| **Cerebrovascular disease** \| 2.15 (1.45-3.18) \| 0.0001 \| 2.17 (1.45-3.25) \| 0.0001 \| \| **Diabetes** \| 1.25 (0.90-1.40) \| 0.2971 \| NA \|  \| \| **Malignancy** \| 0.92 (0.64-1.32) \| 0.666 \| NA \|  \| \| **Sepsis** \| 0.72 (0.58-0.88) \| 0.0022 \| 0.76 (0.61-0.95) \| 0.0168 \| \| **Elective admission** \| 1.32 (0.97-1.80) \| 0.0754 \| 1.25 (0.72-1.47) \| 0.7384 \| \| **Weekend admission** \| 1.01 (0.79-1.28) \| 0.9389 \| NA \|  \| \| **Teaching hospital** \| 1.01 (0.79-1.25) \| 0.9934 \| NA \|  \| \| **Number of organ failures** \| 0.89 (0.82-0.95) \| 0.0015 \| 0.92 (0.85-0.99) \| 0.0366 \| \| **Shockable rhythm** \| 0.95 (0.64-1.41) \| 0.8182 \| NA \|  \| \| **Year of admission** \| 0.97 (0.92-1.03) \| 0.4584 \| NA \|  \| |  |  |  |  |  |  |  |  |  |
|  |  |  |  |  | |  |  |  |  |

**References**

1. Texas inpatient public use data file. Texas Department of State Health Services, Center for Health Statistics, Austin, Texas. Available from: <http://www.dshs.state.tx.us/thcic/hospitals/Inpatientpudf.shtm>.
2. Oud L, Watkins P. [Contemporary trends of the epidemiology, clinical characteristics, and resource utilization of necrotizing fasciitis in Texas: a population-based cohort study.](https://www.ncbi.nlm.nih.gov/pubmed/25893115) Crit Care Res Pract. 2015;2015:618067.
3. Schmidt ML, Barritt AS, Orman ES, Hayashi PH. [Decreasing mortality among patients hospitalized with cirrhosis in the United States from 2002 through 2010.](https://www.ncbi.nlm.nih.gov/pubmed/25623044) Gastroenterology 2015;148:967-977.
4. Singal AK, Salameh H, Kamath PS. [Prevalence and in-hospital mortality trends of infections among patients with cirrhosis: a nationwide study of hospitalised patients in the United States.](https://www.ncbi.nlm.nih.gov/pubmed/24832591) Aliment Pharmacol Ther. 2014;40:105-112.
5. Kramer JR, Davila JA, Miller ED, Richardson P, Giordano TP, El-Serag HB. [The validity of viral hepatitis and chronic liver disease diagnoses in Veterans Affairs administrative databases.](https://www.ncbi.nlm.nih.gov/pubmed/17996017) Aliment Pharmacol Ther. 2008;27:274-282.
6. Stapleton RD, Ehlenbach WJ, Deyo RA, Curtis JR. [Long-term outcomes after in-hospital CPR in older adults with chronic illness.](https://www.ncbi.nlm.nih.gov/pubmed/25086252) Chest. 2014;146:1214-1225.
7. Mallikethi-Reddy S, Briasoulis A, Akintoye E, Jagadeesh K, Brook RD, Rubenfire M, Afonso L, Grines CL. [Incidence and Survival After In-Hospital Cardiopulmonary Resuscitation in Nonelderly Adults: US Experience, 2007 to 2012.](https://www.ncbi.nlm.nih.gov/pubmed/28193738) Circ Cardiovasc Qual Outcomes. 2017;10: e003194.
8. Rhee C, Dantes R, Epstein L, Murphy DJ, Seymour CW, Iwashyna TJ, Kadri SS, Angus DC, Danner RL, Fiore AE, Jernigan JA, Martin GS, Septimus E, Warren DK, Karcz A, Chan C, Menchaca JT, Wang R, Gruber S, Klompas M; CDC Prevention Epicenter Program. [Incidence and Trends of Sepsis in US Hospitals Using Clinical vs Claims Data, 2009-2014.](https://www.ncbi.nlm.nih.gov/pubmed/28903154) JAMA 2017;318:1241-1249.
9. Kazaure HS, Roman SA, Sosa JA. [Epidemiology and outcomes of in-hospital cardiopulmonary resuscitation in the United States, 2000-2009.](https://www.ncbi.nlm.nih.gov/pubmed/23470471) Resuscitation 2013;84:1255-1260.
10. Quan H, Li B, Couris CM, Fushimi K, Graham P, Hider P, Januel JM, Sundararajan V. [Updating and validating the Charlson comorbidity index and score for risk adjustment in hospital discharge abstracts using data from 6 countries.](https://www.ncbi.nlm.nih.gov/pubmed/21330339) Am J Epidemiol 2011;173:676-682.
11. Mallikethi-Reddy S, Briasoulis A, Akintoye E, Jagadeesh K, Brook RD, Rubenfire M, Afonso L, Grines CL. [Incidence and Survival After In-Hospital Cardiopulmonary Resuscitation in Nonelderly Adults: US Experience, 2007 to 2012.](https://www.ncbi.nlm.nih.gov/pubmed/28193738) Circ Cardiovasc Qual Outcomes 2017;10: e003194.
12. Shahreyar M, Dang G, Waqas Bashir M, Kumar G, Hussain J, Ahmad S, Pandey B, Thakur A, Bhandari S, Thandra K, Sra J, Tajik AJ, Jahangir A. [Outcomes of In-Hospital Cardiopulmonary Resuscitation in Morbidly Obese Patients.](https://www.ncbi.nlm.nih.gov/pubmed/29759391) JACC Clin Electrophysiol 2017;3:174-183.
13. Martin GS, Mannino DM, Eaton S, Moss M. [The epidemiology of sepsis in the United States from 1979 through 2000.](https://www.ncbi.nlm.nih.gov/pubmed/12700374) N Engl J Med 2003; 348: 1546-1554.
14. [Bingold TM](https://www.ncbi.nlm.nih.gov/pubmed/?term=Bingold%20TM%5BAuthor%5D&cauthor=true&cauthor_uid=26241475), [Lefering R](https://www.ncbi.nlm.nih.gov/pubmed/?term=Lefering%20R%5BAuthor%5D&cauthor=true&cauthor_uid=26241475), [Zacharowski K](https://www.ncbi.nlm.nih.gov/pubmed/?term=Zacharowski%20K%5BAuthor%5D&cauthor=true&cauthor_uid=26241475), [Meybohm P](https://www.ncbi.nlm.nih.gov/pubmed/?term=Meybohm%20P%5BAuthor%5D&cauthor=true&cauthor_uid=26241475), [Waydhas C](https://www.ncbi.nlm.nih.gov/pubmed/?term=Waydhas%20C%5BAuthor%5D&cauthor=true&cauthor_uid=26241475), [Rosenberger P](https://www.ncbi.nlm.nih.gov/pubmed/?term=Rosenberger%20P%5BAuthor%5D&cauthor=true&cauthor_uid=26241475), [Scheller B](https://www.ncbi.nlm.nih.gov/pubmed/?term=Scheller%20B%5BAuthor%5D&cauthor=true&cauthor_uid=26241475); [DIVI Intensive Care Registry Group](https://www.ncbi.nlm.nih.gov/pubmed/?term=DIVI%20Intensive%20Care%20Registry%20Group%5BCorporate%20Author%5D). Individual Organ Failure and Concomitant Risk of Mortality Differs According to the Type of Admission to ICU - A Retrospective Study of SOFA Score of 23,795 Patients. [PLoS One](https://www.ncbi.nlm.nih.gov/pubmed/26241475) 2015;10: e0134329.
15. Foreman MG, Mannino DM, Moss M. [Cirrhosis as a risk factor for sepsis and death: analysis of the National Hospital Discharge Survey.](https://www.ncbi.nlm.nih.gov/pubmed/12970032)

Chest. 2003 Sep;124(3):1016-20.

1. Wong F, Bernardi M, Balk R, Christman B, Moreau R, Garcia-Tsao G, Patch D, Soriano G, Hoefs J, Navasa M; International Ascites Club. [Sepsis in cirrhosis: report on the 7th meeting of the International Ascites Club.](https://www.ncbi.nlm.nih.gov/pubmed/15831923) Gut. 2005;54:718-725.
2. Trebicka J. Predisposing factors in acute-on-chronic liver failure. Semin Liver Dis 2016;36:167–173.
3. Liu V, Escobar GJ, Greene JD, Soule J, Whippy A, Angus DC, Iwashyna TJ. [Hospital deaths in patients with sepsis from 2 independent cohorts.](https://www.ncbi.nlm.nih.gov/pubmed/24838355) JAMA 2014;312:90-92.
4. Angus DC, Linde-Zwirble WT, Lidicker J, Clermont G, Carcillo J, Pinsky MR. [Epidemiology of severe sepsis in the United States: analysis of incidence, outcome, and associated costs of care.](https://www.ncbi.nlm.nih.gov/pubmed/11445675) Crit Care Med 2001;29:1303-1310.

# [Meyer N](https://www.ncbi.nlm.nih.gov/pubmed/?term=Meyer%20N%5BAuthor%5D&cauthor=true&cauthor_uid=29474320), [Harhay MO](https://www.ncbi.nlm.nih.gov/pubmed/?term=Harhay%20MO%5BAuthor%5D&cauthor=true&cauthor_uid=29474320), [Small DS](https://www.ncbi.nlm.nih.gov/pubmed/?term=Small%20DS%5BAuthor%5D&cauthor=true&cauthor_uid=29474320), [Prescott HC](https://www.ncbi.nlm.nih.gov/pubmed/?term=Prescott%20HC%5BAuthor%5D&cauthor=true&cauthor_uid=29474320), [Bowles KH](https://www.ncbi.nlm.nih.gov/pubmed/?term=Bowles%20KH%5BAuthor%5D&cauthor=true&cauthor_uid=29474320), [Gaieski DF](https://www.ncbi.nlm.nih.gov/pubmed/?term=Gaieski%20DF%5BAuthor%5D&cauthor=true&cauthor_uid=29474320), [Mikkelsen ME](https://www.ncbi.nlm.nih.gov/pubmed/?term=Mikkelsen%20ME%5BAuthor%5D&cauthor=true&cauthor_uid=29474320). Temporal Trends in Incidence, Sepsis-Related Mortality, and Hospital-Based Acute Care After Sepsis. [Crit Care Med](https://www.ncbi.nlm.nih.gov/pubmed/?term=meyer+n+AND+2018+AND+temporal) 2018;46:354-360.

1. Armitage P, Berry G, Matthews JNS. Statistical methods in medical research. 4^th^ ed. Blackwell Science, 2002.
